# Supplementary material for: Lost by Transcription: Fork Failures, Elevated Expression, and Clinical Consequences Related to Deletions in Metastatic Colorectal Cancer
Source: Int J Mol Sci. 2022 May 3;23(9):5080. doi: 10.3390/ijms23095080 (PMC9102808; doi:10.3390/ijms23095080)
Supplement: Supplementary file 1 [file ijms-23-05080-s001.zip › SupplFigures_SVsizes.pptx]

## Slide 1
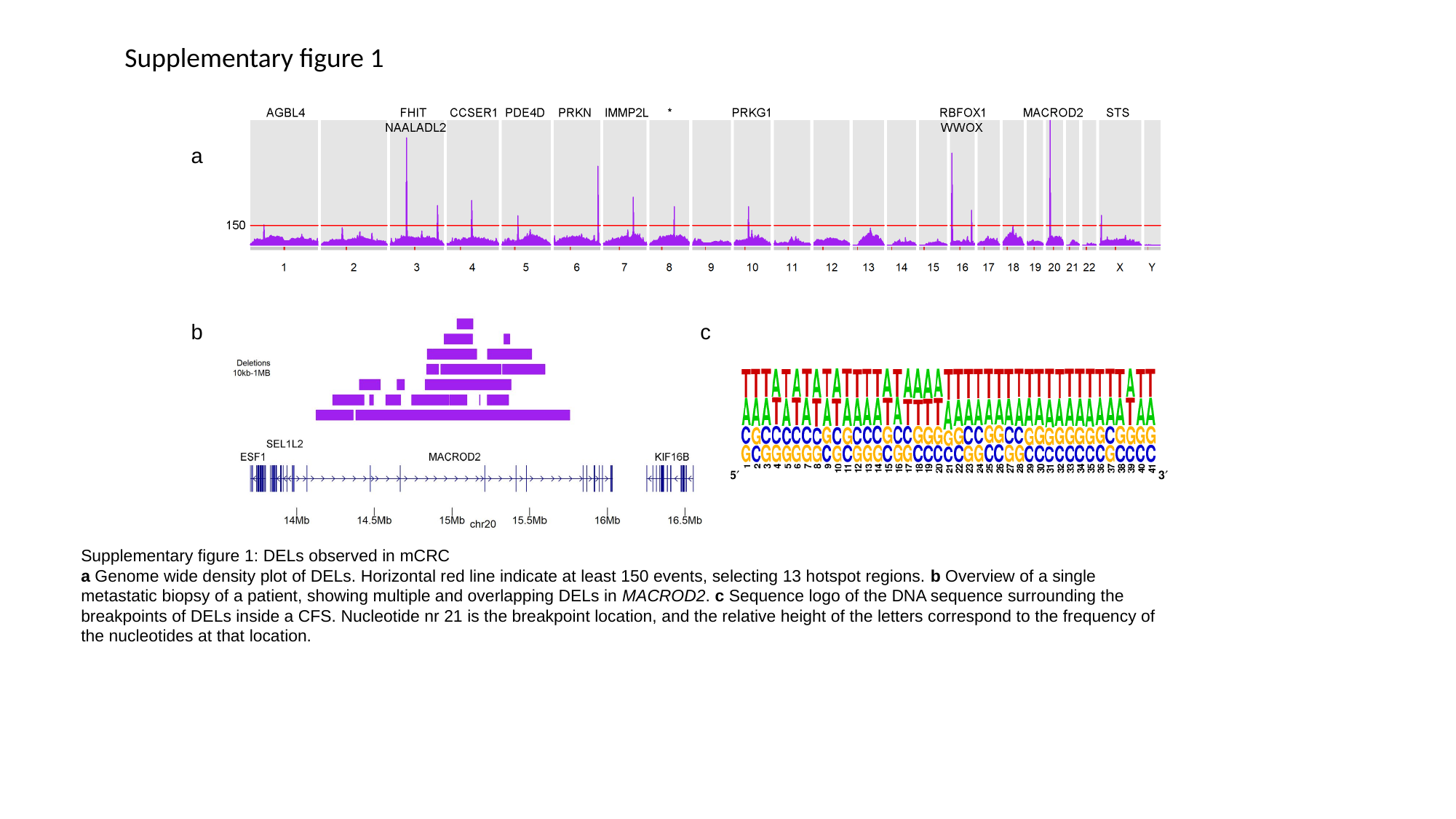

Supplementary figure 1
a
b
c
Supplementary figure 1: DELs observed in mCRC
a Genome wide density plot of DELs. Horizontal red line indicate at least 150 events, selecting 13 hotspot regions. b Overview of a single metastatic biopsy of a patient, showing multiple and overlapping DELs in MACROD2. c Sequence logo of the DNA sequence surrounding the breakpoints of DELs inside a CFS. Nucleotide nr 21 is the breakpoint location, and the relative height of the letters correspond to the frequency of the nucleotides at that location.

## Slide 2
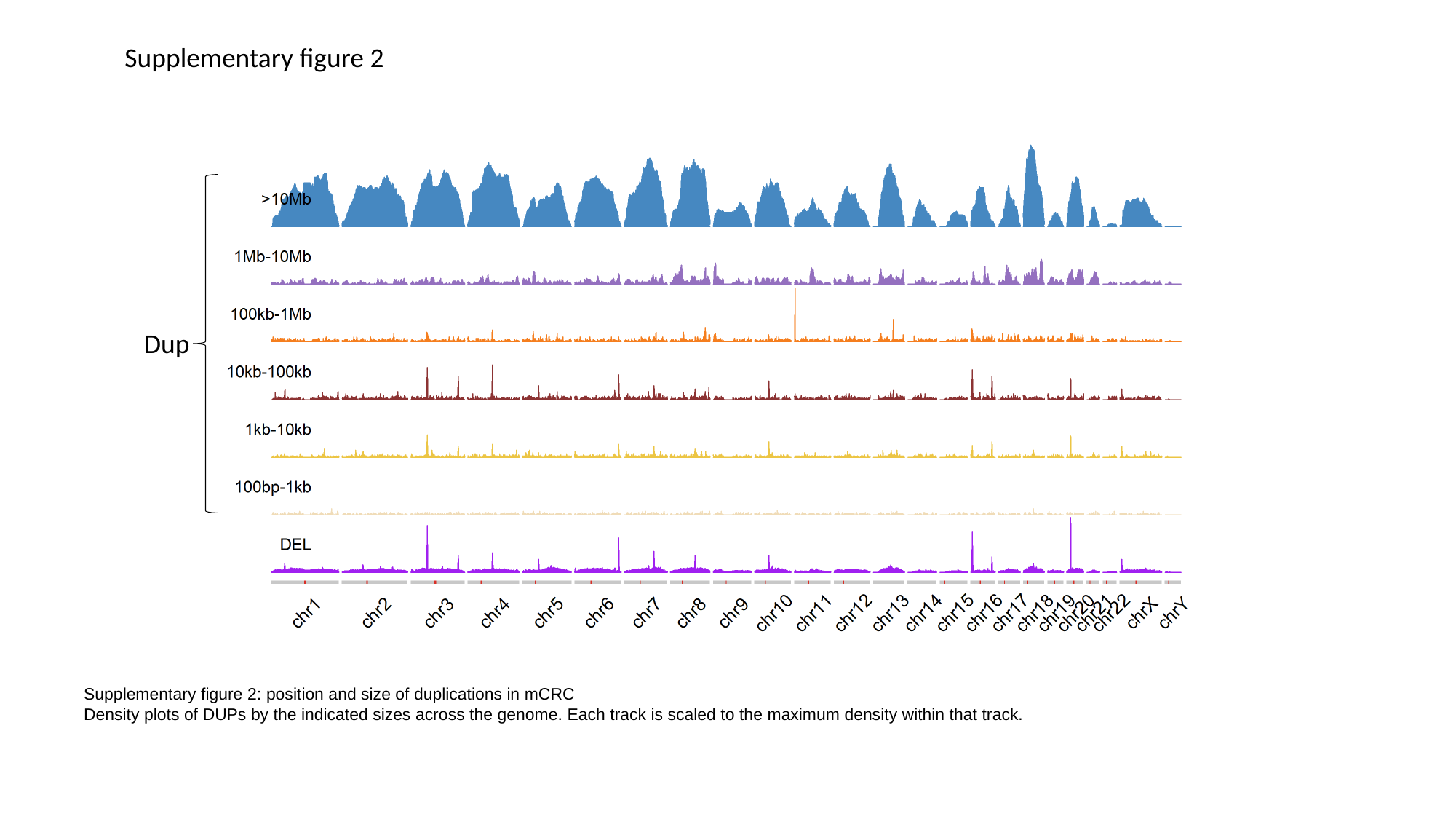

Supplementary figure 2
Dup
Supplementary figure 2: position and size of duplications in mCRC
Density plots of DUPs by the indicated sizes across the genome. Each track is scaled to the maximum density within that track.

## Slide 3
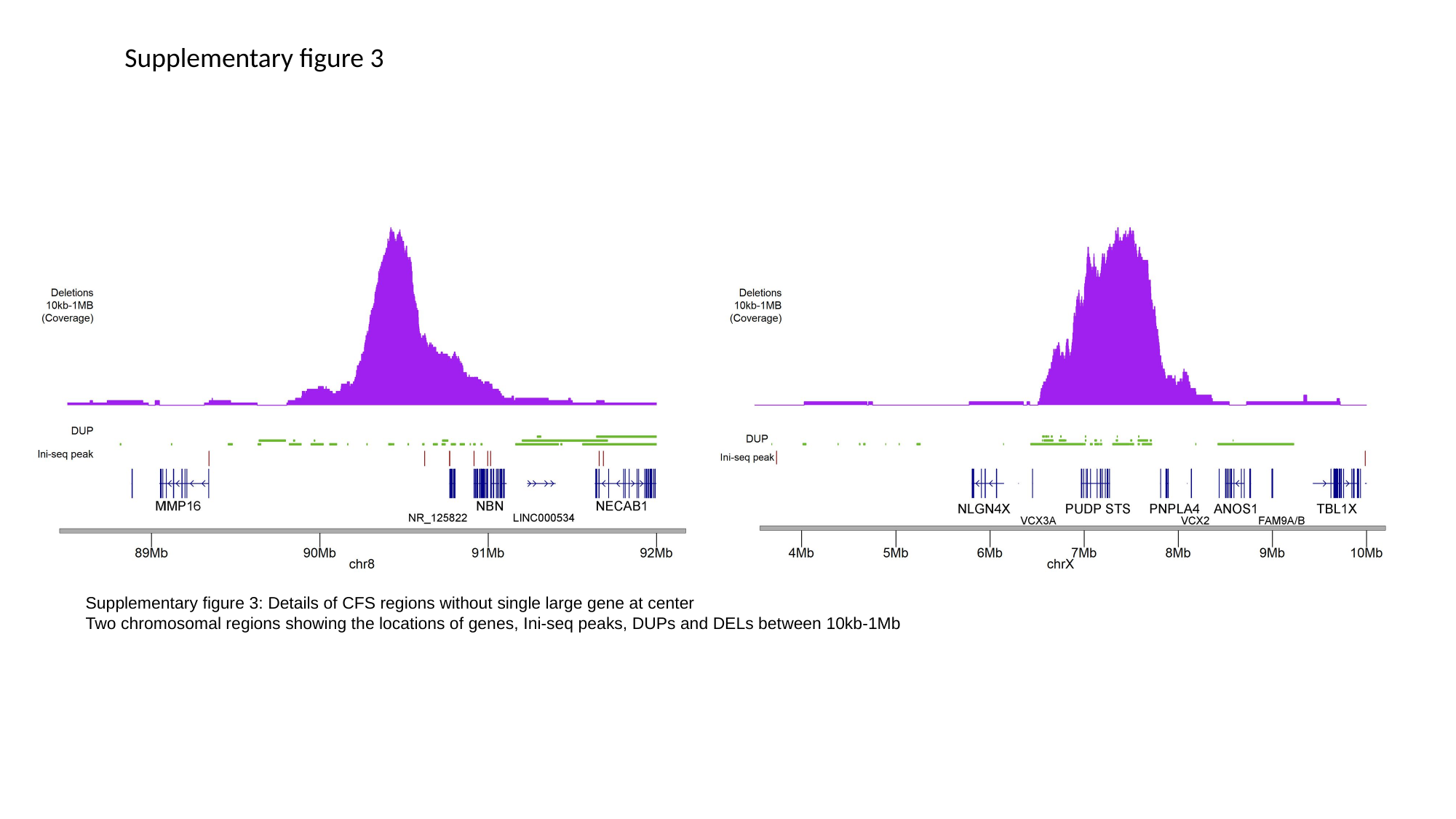

Supplementary figure 3
Supplementary figure 3: Details of CFS regions without single large gene at center
Two chromosomal regions showing the locations of genes, Ini-seq peaks, DUPs and DELs between 10kb-1Mb

## Slide 4
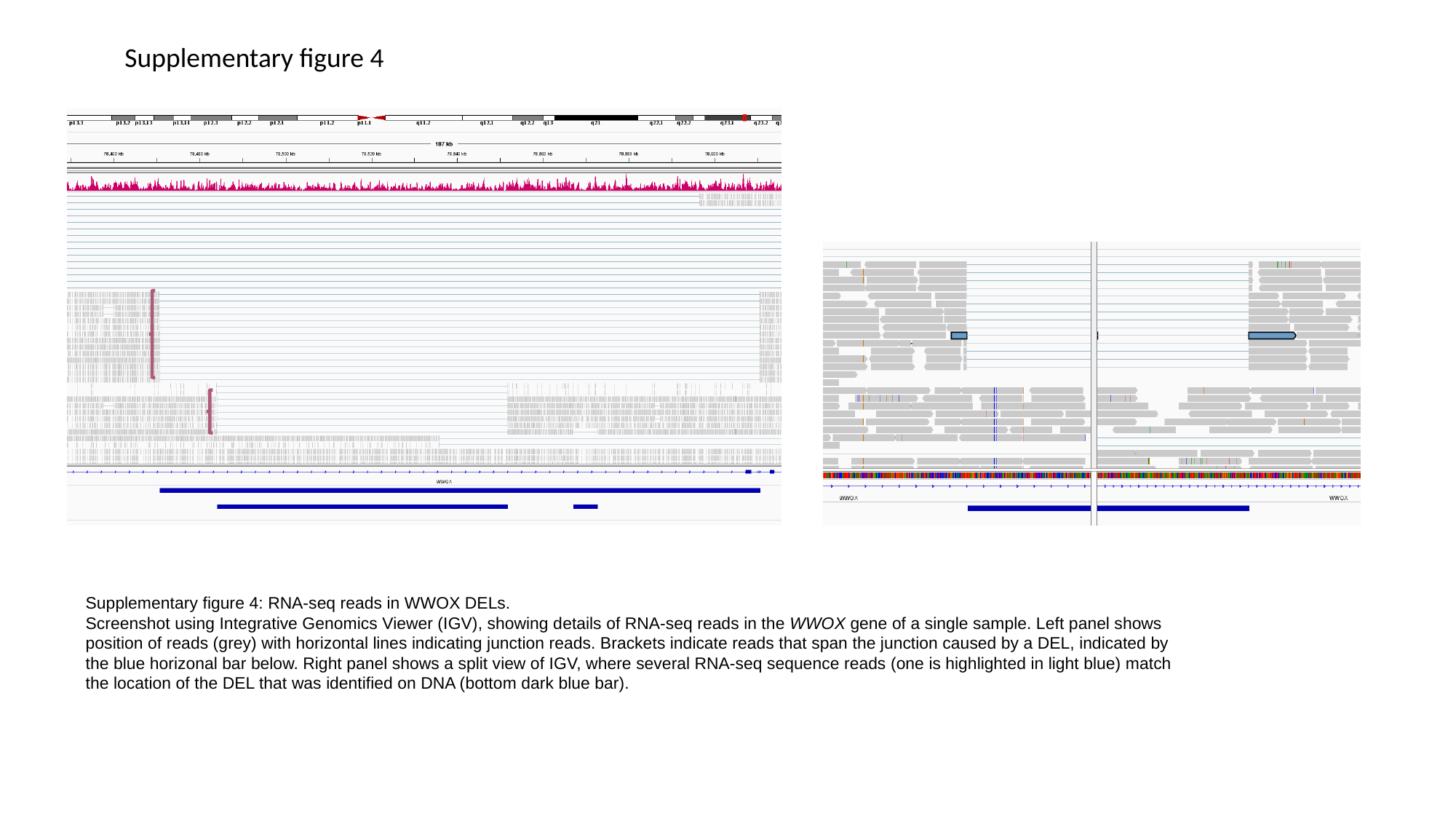

Supplementary figure 4
Supplementary figure 4: RNA-seq reads in WWOX DELs.
Screenshot using Integrative Genomics Viewer (IGV), showing details of RNA-seq reads in the WWOX gene of a single sample. Left panel shows position of reads (grey) with horizontal lines indicating junction reads. Brackets indicate reads that span the junction caused by a DEL, indicated by the blue horizonal bar below. Right panel shows a split view of IGV, where several RNA-seq sequence reads (one is highlighted in light blue) match the location of the DEL that was identified on DNA (bottom dark blue bar).

## Slide 5
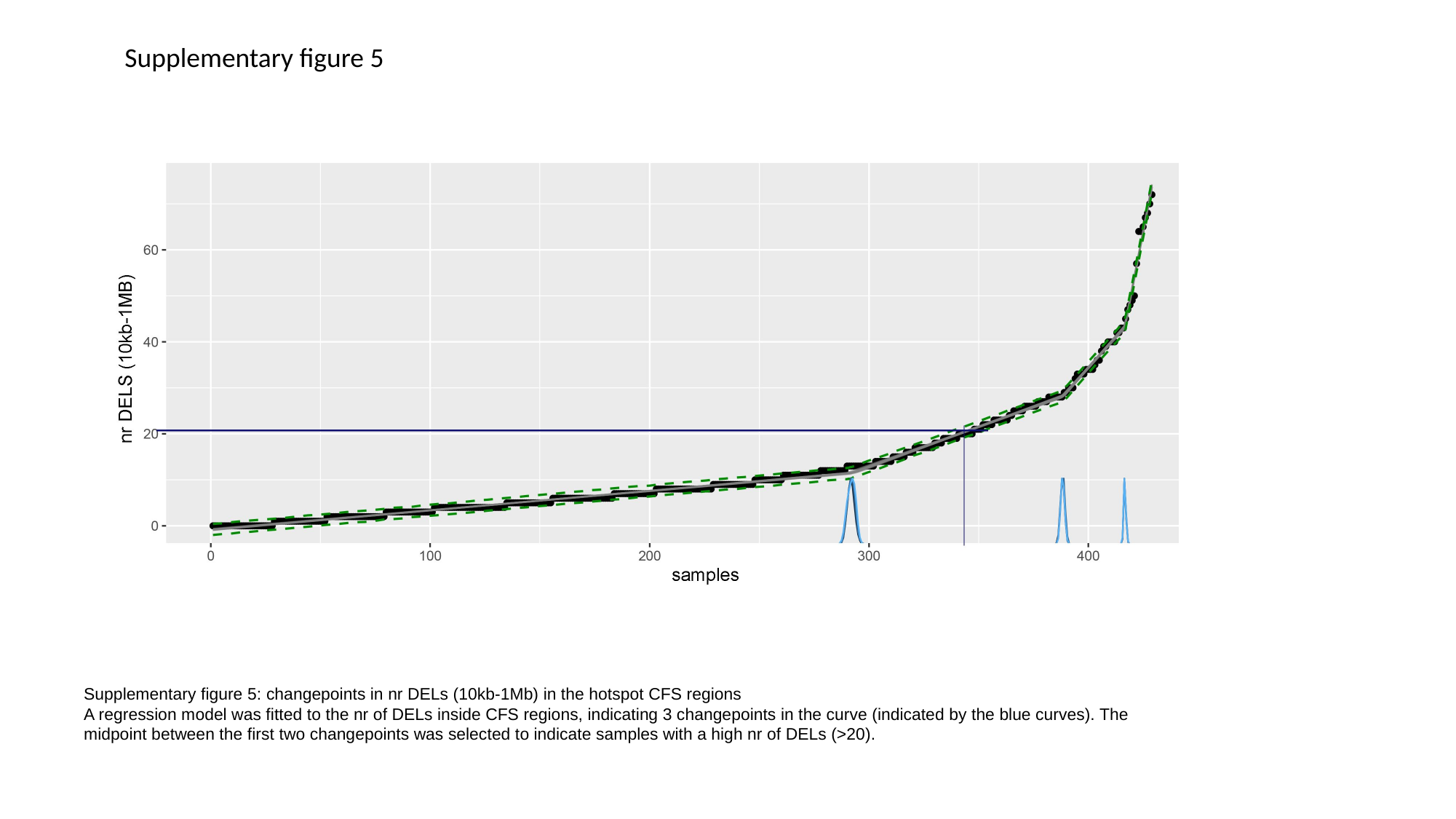

Supplementary figure 5
Supplementary figure 5: changepoints in nr DELs (10kb-1Mb) in the hotspot CFS regions
A regression model was fitted to the nr of DELs inside CFS regions, indicating 3 changepoints in the curve (indicated by the blue curves). The midpoint between the first two changepoints was selected to indicate samples with a high nr of DELs (>20).
